# Mayer-Rokitansky-Küster-Hauser (MRKH) syndrome: a comprehensive update

**DOI:** 10.1186/s13023-020-01491-9

**Published:** 2020-08-20

**Authors:** Morten Krogh Herlin, Michael Bjørn Petersen, Mats Brännström

**Affiliations:** 1 Department of Clinical Genetics, Aalborg University Hospital, Aalborg, Denmark; 2 Department of Clinical Genetics, Aarhus University Hospital, Brendstrupgårdsvej 21C, DK-8200 Aarhus N, Denmark; 3 Department of Clinical Medicine, Aalborg University, Aalborg, Denmark; 4 Department of Obstetrics and Gynecology, Sahlgrenska Academy, Gothenburg, Sweden

**Keywords:** MRKH syndrome, MRKHS, Disorders of sex development, 46,XX DSD, Female infertility, Female genitalia, Müllerian aplasia, Vaginal agenesis, Uterus transplantation, Genetics

## Abstract

**Background:**

Mayer-Rokitansky-Küster-Hauser (MRKH) syndrome, also referred to as Müllerian aplasia, is a congenital disorder characterized by aplasia of the uterus and upper part of the vagina in females with normal secondary sex characteristics and a normal female karyotype (46,XX).

**Main body:**

The diagnosis is often made during adolescence following investigations for primary amenorrhea and has an estimated prevalence of 1 in 5000 live female births. MRKH syndrome is classified as type I (isolated uterovaginal aplasia) or type II (associated with extragenital manifestations). Extragenital anomalies typically include renal, skeletal, ear, or cardiac malformations. The etiology of MRKH syndrome still remains elusive, however increasing reports of familial clustering point towards genetic causes and the use of various genomic techniques has allowed the identification of promising recurrent genetic abnormalities in some patients. The psychosexual impact of having MRKH syndrome should not be underestimated and the clinical care foremost involves thorough counselling and support in careful dialogue with the patient. Vaginal agenesis therapy is available for mature patients following therapeutical counselling and education with non-invasive vaginal dilations recommended as first-line therapy or by surgery. MRKH syndrome involves absolute uterine factor infertility and until recently, the only option for the patients to achieve biological motherhood was through gestational surrogacy, which is prohibited in most countries. However, the successful clinical trial of uterus transplantation (UTx) by a Swedish team followed by the first live-birth in September, 2014 in Gothenburg, proofed the first available fertility treatment in MRKH syndrome and UTx is now being performed in other countries around the world allowing women with MRKH syndrome to carry their own child and achieve biological motherhood.

**Conclusion:**

Several advances in research across multiple disciplines have been made in the recent years and this kaleidoscopic review provides a current status of various key aspects in MRKH syndrome and provides perspectives for future research and improved clinical care.

## Background

**Fig. 1:**
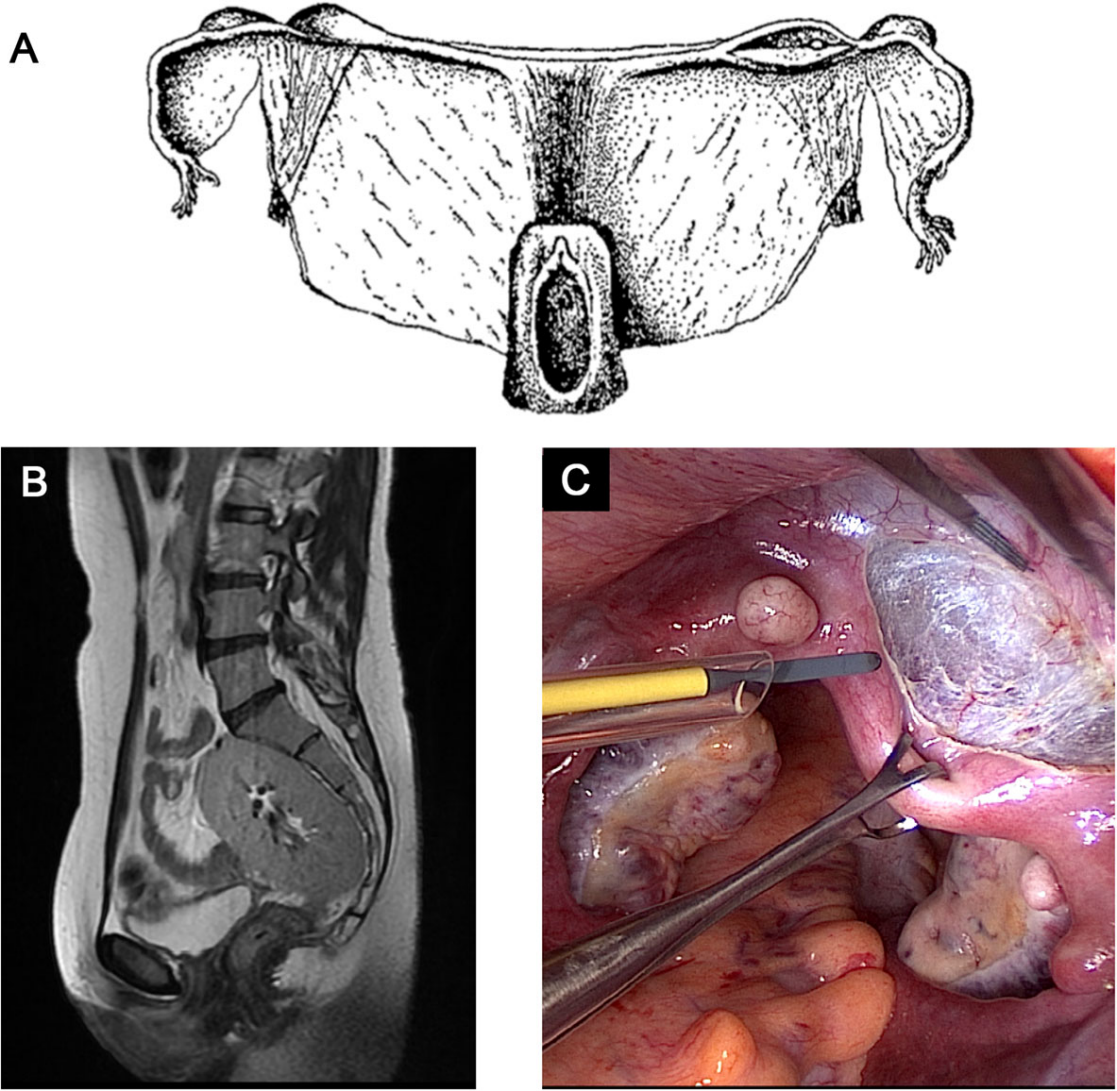
**a** The original illustration by Carl von Rokitansky (1838) showing the uterovaginal morphology in MRKH syndrome with a shortened blind-ending vagina and two rudimentary uterine remnants. **b** A sagittal T2-weighted MRI showing complete uterovaginal absence in type II MRKH syndrome associated with renal agenesis and a solitary pelvic kidney. **c** The pelvis of a patient with MRKH syndrome during surgical preparation for uterus transplantation. The forceps is holding the fibrous uterine rudiment in the midline, while it is dissected free from the bladder. On the uterine buds, located on the pelvic sidewalls, small subserosal leiomyomas are seen on both sides. The ovaries are located medially towards the Pouch of Douglas

Mayer-Rokitansky-Küster-Hauser (MRKH) syndrome [ICD-10 Q51.0/Q52.0; OMIM %277000/%601076; ORPHA 3109], also referred to as Müllerian aplasia or congenital absence of the uterus and vagina, is a congenital disorder characterized by agenesis or aplasia of the uterus and upper part of the vagina in females with a normal female karyotype (46,XX) (Fig. 1). The external genitalia appear normal and the patients typically have a normal reproductive endocrine function and reach puberty showing normal signs of thelarche and pubarche. Patients typically present with primary amenorrhea during adolescence and MRKH syndrome has been reported in ~ 16% of patients with primary amenorrhea, thus considered the second most common cause hereof after ovarian failure [1]. Müllerian aplasia can be associated with extragenital malformations involving mainly the kidneys and skeleton.

During the last decade several advances have been made in MRKH syndrome research, especially within the fields of genetics, non-surgical management, and uterus transplantation as the first available fertility treatment. Herein, we provide a detailed kaleidoscopic review of key aspects on MRKH syndrome including the most recent advances in the field.

## Main text

### Epidemiology

The prevalence of MRKH syndrome is generally considered to be in 1 in 5000 live female births but it remains poorly investigated [2, 3]. The first reported prevalence was on vaginal agenesis by John Engstad in 1917, where he suggested a 1 in 5000 prevalence based on nine patients in his clinic [4]. Other estimates range from 1 in 4000-20,000. In 1942, Owens found six patients in 125,000 admissions [5]. Bryan et al. in 1949 reported a prevalence of 1 in 4000 females based on 100 patients with vaginal agenesis [6]. Later in 1981, Evans et al. reported the first population-based prevalence in the state of Michigan being 1 in 10,588 females. It is important to consider that perhaps not all patients in these cohorts had MRKH syndrome as other diagnoses may also present with vaginal agenesis. To date, only two population-based studies investigating the prevalence of MRKH syndrome have been published. Aittomäki et al. reported on a cohort of 161 patients treated at the five university hospitals in Finland from 1978 to 1993. Seventy-seven of the patients, born from 1960 to 1969, were included in the prevalence estimate of 1 in 4961 newborn girls [2]. Recently, Herlin et al. reported on a cohort in Denmark identified through searches in the National Danish Patient Registry from 1994 to 2015 followed by medical chart reviews. From the cohort of 168 patients, 138 patients born from 1974 to 1996 were included and resulted in a prevalence of 1 in 4982 (95% confidence interval: 4216-5887) live female births [3]. The two studies were based on European populations and thus, it remains unknown whether the prevalence in other populations differs.

### History and classification

MRKH syndrome is named after the authors of the four original descriptions published over a 130 year period by German anatomist August Franz Josef Karl Mayer (1829), Austrian anatomist Carl von Rokitansky (1838; Fig. 1a), German gynecologist Hermann Küster (1910) and Swiss gynecologist Georges Andre Hauser (1961) [7–10]. The first description of congenital absence of the uterus and vagina, however, is attributed to the Italian anatomist Realdo Colombo although his descriptions lacked detail. In his main work *De Re Anatomica* from 1562*,* he described in volume 15 titled *De iis quae raro in anatome reperiuntur* (eng. “rare findings in anatomy”) an entity he named *vulva rara* in a woman with no womb and vagina who complained of pain upon coitus [11]. Centuries after Mayer and Rokitansky independently reported on autopsies of two deceased women in which they identified rudimentary uterine buds and described what they referred to as *uterus bipartitus solidus* cum *vagina solida* [7, 8]. Küster was the first to report this finding in a living patient from whom he removed the pain-causing uterine remnants [9], whereas Hauser and Schreiner completed the definition of uterovaginal agenesis in women with normal secondary sex characteristics and normal female karyotype based on a series of 21 cases [10].

These contributions all described isolated uterovaginal agenesis with no associated extragenital malformations, which in contemporary literature is referred to as type I MRKH syndrome. In 1977, Schmid-Tannwald and Hauser described seven cases with renal malformations which was named atypical MRKH syndrome [12]. Duncan et al. reported two cases with a severe phenotype of uterovaginal agenesis associated with renal and skeletal malformations and suggested the entity MURCS association (Müllerian duct aplasia, renal aplasia and cervicothoracic somite dysplasia). In current literature these two subgroups are grouped together as type II MRKH syndrome referring to all cases with any associated extragenital abnormality (renal, skeletal, and others). The frequency of type I and type II MRKH syndrome is 56–72% and 28–44%, respectively [3, 13–15].

### Embryology, etiology, and genetics

The female reproductive tract in humans includes the oviducts (Fallopian tubes), uterus, cervix and vagina. The oviducts, uterus, cervix, and upper two-thirds of the vagina origin from the paramesonephric (Müllerian) ducts (PMD), whereas the lower part of the vagina origins from the urogenital sinus [16]. Formation of the PMD starts around 5th–6th gestational week as bilateral craniocaudal invaginations of the coelomic epithelium of the urogenital ridges (intermediate mesoderm) growing caudally guided by the mesonephric (Wolffian) ducts to reach the urogenital sinus (endoderm) [17]. The caudal part of the two PMDs fuses to form the uterus, cervix and upper vagina, whereas the upper parts of the PMDs form the two oviducts. MRKH syndrome is caused by either complete agenesis or aplasia of the PMDs to form the uterus and upper vagina.

The etiology of MRKH syndrome still remains unclear. Tissue patterning and organ morphogenesis in the human embryo is a complex process and result from a combination of timely cues from genetic factors, soluble morphogens, chemical factors and mechanical forces [18] and several possible etiologies should therefore be considered at this current state of knowledge including monogenic, oligogenic, polygenic, multifactorial, and environmental factors. Moreover, regulatory mechanisms (e.g. epigenetic factors) and somatic genetic events during development could also be involved. Increasing reports of familial occurrence of MRKH syndrome and its associated anomalies support a monogenic genetic etiology [19, 20]. Most pedigrees suggest autosomal dominant inheritance with incomplete penetrance and this especially seems to include families with aggregation of both MRKH syndrome and renal abnormalities which in previous literature has been referred to as hereditary urogenital adysplasia [19, 21–24]. In contrast, most cases occurring sporadically, lacking recurrence in outcomes of surrogate pregnancies [25, 26] and several reports of discordant twin pairs [3, 27–30] support either polygenic/multifactorial or non-genetic etiologies (e.g. teratogenic exposures in utero [31]). Discussing possible etiologies for MRKH syndrome and the predominance of mainly sporadic cases, however, it should be remembered that the nature of MRKH syndrome involving absolute uterine factor infertility hinders vertical transmission of the trait and thus, the genetic contribution in MRKH syndrome may therefore be underestimated.

Early genetic studies used a hypothesis-based candidate gene approach. In male embryos, anti-Müllerian hormone (AMH) inhibits the development of Müllerian structures, which led to idea of overexpression of AMH and its receptors as a cause of MRKH syndrome. However, studies have failed to find evidence for this hypothesis [32–34]. Several other candidate genes based on developmental pathways and associated diseases have disproved to cause MRKH syndrome [35–48]. Mutations in *WNT4* were detected in patients with Müllerian aplasia and virilization/hyperandrogenism [49–51]. However, this should be considered a separate entity and not a cause of MRKH syndrome (OMIM #158330) [52–54]. Furthermore, genetic variants have been reported in the *WNT9B* gene, involved in genitourinary development acting upstream of *WNT4* [55, 56]. Recently, a study investigated male microchimerism as a possible cause but again without finding evidence to support this [57].

Several interesting genetic findings emerged following the advent use of chromosomal microarray. This method enabled hypothesis-free genome-wide searches for chromosomal imbalances (deletions/duplications) and several recurrent copy number variations (CNV) have been identified located at chromosomal regions 1q21.1, 16p11.2, 17q12, and 22q11.21 (summarized Table 1) [20, 44, 58–63, 65–67]. Still, it is important to be cautious concluding these findings as confirmed pathogenic lesions. The most promising genetic CNVs are 17q12 and 16p11.2. The 17q12 locus encompasses *LHX1* and *HNF1B*. Single nucleotide variants in *LHX1* have been reported in MRKH syndrome [59, 71] and *Lim1* knock-out in mice results in a Müllerian aplasia [68]. Variants in *HNF1B* have been associated with various renal and uterine abnormalities [75, 76], however its exact role in MRKH syndrome pathogenesis is uncertain. The 16p.11.2 locus encompasses the *TBX6* gene in which single nucleotide variants of unknown significance have also been reported [55, 63, 69, 70].

**Table 1:** Recurrent genetic abnormalities associated with MRKH syndrome

***Copy number variations (chromosomal imbalances)***						
Chromosome location	Imbalance	# of CNVs reported	Size range	Suspected genes involved	Phenotype^a^	Ref.
1q21	Deletion	3	0.4–4 Mb	*RBM8A*	Type I + II	[20, 58–60]
	Duplication	2	0.26–2.7 Mb			
16p11.2	Deletion	10	0.5–0.7 Mb	*TBX6*	Type I + II	[20, 61–64]
17q12	Deletion	13	1.2–1.9 Mb	*LHX1, HNF1B*	Type I + II	[20, 58–60, 62, 63, 65, 66]
22q11	Deletion	4	0.39–5.24 Mb	Uncertain (*TBX1* not involved in all CNVs)	Type I + II	[44, 58, 59, 62, 65, 67]
	Duplication	1	3.5 Mb			
***Single nucleotide variations***						
Chromosome location	Gene	Variant^b^		Gene function / validation	Phenotype	Ref.
16p11.2	*TBX6*	c.484G > A; p.Gly162Ser		The gene encodes a transcription factor involved in mesoderm formation and specification [68]	Type I + II	[63, 69, 70]
		c.622-2A > T; splice variant				
		c.815G > A; p.Arg272Gln				
		c.1146C > A; p.Tyr382*				
17q12	*LHX1*	c.11G > C; p.Cys4Ser		*Lim1*-null mice also have MA [63]	Type I	[59, 63, 71]
		c.25dup; p.Arg9Lysfs*25				
		c.790C > G; p.Arg264Gly				
		c.935C > A; p.Pro312His				
		c.995C > G; p.Arg332Pro				
17q21.32	*WNT9B*	c.472C > G; p.Gln158Glu		*Wnt9b-*null mice also have MA. *Wnt9b* acts upstream of *Wnt4* [53].	Type I	[55, 56]
		c.665G > A; p.Arg222His				
		c.722G > A; p.Arg241His				
		c.974G > A; p.Arg325His				
		c.1029C > A; p.Cys343*				
18q11.1–2	*GREB1L*	c.705G > T; p.Trp235Cys		Additional *GREB1L* variants of unknown significance have been reported in unrelated MRKH syndrome patients [72]. Several *GREB1L* variants have been reported in female fetuses with BRA and uterus/uterovaginal agenesis [72–74]. *Greb1l-*null mice also have BRA and Müllerian aplasia [73, 74].	Type II with renal malfor-mations (familial, autosomal dominant inheritance)	[24, 72]
		c.2227del; p.(Gln743Argfs*10)				

*Abbrevations*: *BRA* bilateral agenesis, *CNV* copy number variations, *MA* Müllerian aplasia, *URA* unilateral renal agenesis

^a^MRKH type II also encompasses MURCS association; ^b^Trancripts for the genes are: *TBX6*, NM_004608.3; *LHX1*, NM_005568.3; *WNT9B*, NM_003396.1; *GREB1L*, NM_001142966.2

Despite the progress following chromosomal microarray, genetic findings reported so far only apply to a minority of patients. During the last decade, the increasing availability of massively parallel sequencing technologies (also referred to as Next-Generation Sequencing, NGS) has provided new optimism in the search for genes implicated in MRKH syndrome. Recently, Pan et al. performed whole-genome sequencing (WGS) analysis of nine MRKH syndrome trios (patient and both parents) demonstrating the capacity of WGS in unbiased detection of de novo genomic variation [77]. Other recent studies also used NGS technology in genomic searches for genetic variation [24, 64, 78, 79]. However, it remains a challenge to interpret the pathogenicity of these findings and investigations of larger cohorts will likely be needed in order to identify recurrent genetic variation. Furthermore, it is essential to obtain a detailed family history and consider radiological examinations for subtle genitourinary anomalies in relatives of the patients. In 2019, Herlin et al. described whole-exome sequencing (WES) in a three-generation family with two female cousins having type II MRKH syndrome and unilateral renal agenesis, and two male relatives with renal agenesis. The study reported a co-segregating missense variant in *GREB1L* (Table 1) [24]*,* a gene identified in 2017 to cause bilateral renal agenesis in fetuses of which several female fetuses also had uterus agenesis [73, 74]. Just recently, Jacquinet et al. reported four additional multiplex families with either type II MRKH syndrome or uterovaginal aplasia (fetuses) associated with renal malformations (agenesis in particular) in which pathogenic *GREB1L* variants were identified from WES data [72]. *GREB1L* now seems to be the first gene to show a strong association with type II MRKH syndrome with kidney anomalies following autosomal dominant inheritance with incomplete penetrance.

Continuous research in the genetics of MRKH syndrome is imperative to provide better knowledge of the pathogenesis and improve the patient care and counselling. However, the anticipated identification of novel monogenic causes of MRKH syndrome brings forward new difficult questions. The increasing availability of uterus transplantation as fertility treatment or in vitro fertilisation (IVF) using a gestational carrier will allow more patients to achieve biological motherhood in the future, and thus we could expect an increasing demand for prenatal diagnostics including preimplantation genetic testing from some patients having a genetic diagnosis for their disorder. Views on prenatal genetic testing are highly individual and some patients will request it, while others will oppose it. Therefore, the reproductive autonomy of each patient should always be respected. Importantly, such options should only be considered for patients with a robust genetic diagnosis with a notable recurrence risk and should always be preceded by thorough genetic counseling.

### Clinical presentation, diagnosis, and associated malformations

Patients with MRKH syndrome typically present during adolescence with primary amenorrhea defined as absent menstrual periods at the age of 16 following normal puberty and development of secondary sex characteristics. Other complaints at referral include dyspareunia/apareunia and (cyclic) abdominal pain. Finally, patients (typically younger children) may be referred after an incidental finding of vaginal or uterus agenesis, but if examined by imaging at young age such findings may be false interpretations of the prepubertal uterus. Median age at referral has been reported to be 17.5 years (interquartile range: 16–19) [3].

Table 2 summarizes the routine diagnostic work-up and typical findings in MRKH syndrome. Patients with primary amenorrhea should be referred to a gynecology department or a gynecologist with expertise in pediatric/adolescent gynecology or disorders in sex development (DSD) for examination. Physical examination is carried out which may include examination of external genitalia and examination of the introitus/vagina with respect to the patient’s age and motivation (should be avoided in prepubertal adolescents). Transperineal or transabdominal ultrasonography (US) is performed revealing the absence of the uterus and presence of ovaries. Magnetic resonance imaging (MRI) of the internal genitalia is considered the golden standard method for the diagnosis of uterovaginal agenesis in MRKH syndrome and should always be performed when available. MRI is non-invasive and superior to computed tomography (CT) in showing the Müllerian structures in detail (uterine remnants or complete agenesis) including the presence of endometrium in uterine remnants. MRI also shows the ovaries and extragenital malformations, and has high interrater agreement with laparoscopy [80, 81]. Thus, laparoscopy is rarely indicated for diagnostic purposes only, but may be relevant in patients with pain-causing uterine remnants where surgical removal of the tissue is needed [82]. Examination of the kidneys by US/MRI should be performed to screen for renal malformations (prevalence of ~ 30%) [3, 81]. Detailed screening of other typical extragenital anomalies (skeletal, ear, cardiac etc.), by imaging and otorhinopharyngeal assessment, is not done routinely but should be considered in case of relevant complaints or findings at the physical examination. More studies are still needed to conclude on the relevance of a full screening of extragenital abnormalities for all patients and its recommendation in clinical practice. Chromosomal analysis by G/Q-banding is often performed to confirm normal female karyotype (46,XX). Chromosomal microarray analysis can be considered for the detection of copy number variations but is not obligate for the diagnosis. Overall test positive rate for imbalances has been reported at ~ 16–20% but the interpretation of the pathogenicity of these findings remains a challenge [20, 61, 63]. Other relevant laboratory tests include FSH, LH, androgens and estradiol, which are generally considered to be normal in MRKH syndrome [3, 83]. However, biochemical (non-clinical) hyperandrogenemia was recently reported in ~ 50% of patients [84], but this finding requires further validation.

**Table 2:** Routine diagnostic work-up in MRKH syndrome

Examination	Typical findings
Physical examination including a precautious pelvic exam by an experienced pediatric/adolescent gynecologist.	Normal height, secondary sex characteristics, and hair growth. Normal external genitalia. Short blind-ending vagina (0–3 cm) with no cervix at the apex. No uterus detected by manual palpation.
Radiologic examination	
US of internal genitalia (transvaginal/−perineal)^a^	No uterus or vaginal canal. Two functional ovaries.
Pelvic MRI scan	Confirms the diagnosis. Determines the presence of rudimentary uterine buds or complete uterovaginal agenesis
Renal scan (by US or MRI)	Renal abnormalities are found in approximately 30% of patients
Consider examinations for other associated malformations (e.g. EOS scan, otorhinopharyngeal assessment and echocardiography	Various skeletal malformations (axis and limbs), hearing impairment and congenital heart defects (rare).
Biochemical analysis	
Gonadotropins (FSH, LH)	Normal levels following menstrual cycle
Estradiol	Normal levels
Androgen status	Normal female levels
Chromosomal analysis (can be used to differentiate from 46,XY DSDs)	46,XX

*Abbreviations*: *FSH* follicle stimulating hormone, *LH* luteinizing hormone, *MRI* magnetic resonance imaging, *US* ultrasonography

^a^Transabdominal US should be considered in younger patients

Uterus agenesis/aplasia in MRKH syndrome has two phenotypic presentations. Two aplastic uterine buds on the pelvic sidewall derived from the Müllerian ducts (often seen in type I) or complete absence of one or both Müllerian ducts (often seen in type II associated with ipsilateral kidney malformations). The two uterine buds are seen in combination with a uterine remnant or fibrous band in the midline (Fig. 1c). Presence of uterine remnants have been reported in 48–95% of the patients [85–89] and is associated with a risk of cyclic (catamenial) abdominal pain due to the presence of active endometrium [85, 87, 90]. Some patients with endometrium that respond to the cyclic steroid changes of the menstrual cycle may even develop hematometra due to cryptomenorrhea in the remnant cavity [91, 92]. In case of cyclic abdominal pain and endometrial activity on MRI, laparoscopy and surgical removal of the uterine remnants containing endometrium should be considered. Endometriosis is another feature which may occur in MRKH syndrome, especially in patients with uterine remnants and active endometrium [85, 93]. This phenomenon in MRKH syndrome is often hypothesized to be explained by retrograde menstruation (Sampson’s implantation theory) which is further supported by the association of endometriosis with other obstructive Müllerian anomalies, e.g. Herlyn-Werner-Wunderlich syndrome [94, 95]. However, it should be noted that the implantation theory does not fit with all endometriotic lesions including endometriosis in MRKH syndrome patients with no uterine remnants and extra-abdominal endometriosis for which other theories are valid [96]. Other gynecological features that might be encountered in MRKH syndrome include leiomyomas in the rudimentary uterus (Fig. 1c) [97], adenomyosis [98] and inguinal ovarian/Müllerian duct hernias [99, 100].

In MRKH syndrome, both ovaries are typically present and well-functioning. However, their anatomical position is usually more cranial than the normal position and they are often found lateral, rather than medial, to the external iliac arteries, probably due to the lack of Fallopian tube development. Ovary anomalies are rare and only found in ~ 5–10% [3, 13, 101]. Different anomalies previously reported include unilateral agenesis, ectopic ovaries, polycystic ovaries, streak ovaries, and rarely tumors [102].

As mentioned above, MRKH syndrome is classified into two groups. Type I (isolated) without any extragenital abnormalities and type II (including MURCS association) with presence of extragenital abnormalities. Table 3 summarizes the extragenital abnormalities reported from larger cohorts [3, 14, 15, 86, 101, 103–105]. Renal malformations are the most frequent extragenital abnormalities in MRKH syndrome occurring in ~ 30–40% in European cohorts. Unilateral renal agenesis (URA) is the most frequent anomaly accounting for around half of all renal malformations associated with MRKH syndrome (Fig. 1b). Notably, URA is often associated with complete absence of the ipsilateral Müllerian duct which suggests a close relationship between early kidney and Müllerian duct development [81]. Other renal malformations include pelvic kidney, duplex kidney, and horseshoe kidney (Table 3). Interestingly, Deng et al. reported a lower prevalence of renal malformations of only 13% in their Chinese cohort suggesting the possibility of inter-ethnic phenotypical variations in MRKH syndrome from European patient cohorts [15]. Pan et al. reported an even lower prevalence of 5% in their Chinese cohort but that study did not include information on the extent of renal examinations performed, which could imply an underestimation of the prevalence [106]. Anomalies of the skeleton are the second most frequent extragenital manifestations in MRKH syndrome affecting around ~ 10–40% depending on the extent of examinations performed and anomalies included (Table 3). Skeletal anomalies typically involve the axial skeleton (e.g. scoliosis, Klippel-Feil anomaly, hemivertebrae, rib aplasia etc.) and more rarely the extremities. Cardiac abnormalities are reported in < 5% of patients (e.g. pulmonary valve stenosis, atrial septal defect). Hearing impairment including both sensorineural and conductive hearing loss (e.g. external meatus atresia, stapedial ankylosis) are generally reported in < 5%, but is not routinely examined. Strübbe et al. performed a systematic evaluation of associated anomalies with otorhinopharyngeal assessment and reported ear abnormalities in 11% of patients, which could indicate an underestimation [107]. In a small number of patients, MRKH syndrome has been reported with a very severe phenotype: vertebral defect, anal atresia, cardiac defect, tracheoesophageal fistula/esophageal atresia, renal defect, and limb defect (VACTERL association), while even fewer have been reported with isolated anorectal malformations [3, 103, 108].

**Table 3:** Classification and extragenital anomalies associated with MRKH syndrome reported in large cohorts (> 100 patients)

Study	Oppelt et al., 2006 [13]		Creatsas et al., 2010 [86]		Oppelt et al., 2012 [101]		Lalatta et al., 2015 [14]		Rall et al., 2015 [103]		Willemsen and Kluivers, 2015 [104]		Kapczuk et al., 2016 [105]		Herlin et al., 2016 [3]		Deng et al., 2019 [15]	
Population	Mixed		Greece		Germany		Italy		Germany		Netherlands		Poland		Denmark		China	
Setting	Literature review		Tertiary center		Two tertiary centers		Tertiary center		Tertiary center		Tertiary center		Tertiary center		Nationwide		Tertiary center	
No. of patients	*n* = 512		*n* = 200		*n* = 284		*n* = 115		*n* = 346		*n* = 254		*n* = 125		*n* = 168		*n* = 274	
Classification																		
Type I (isolated)	333	(64%)	NS		156	(55%)	83	(72%)	184	(53%)	160	(69%)^b^	57	(46%)	65	(57%)^d^	197	(72%)
Type II (syndromic)	127	(36%)	NS		126	(44%)	32	(28%)	162	(47%)	72	(31%)	68	(54%)	50	(43%)	77	(28%)
MURCS association	61	(12%)	NS		NS		6	(6%)	19	(5%)	58	(25%)	52	(42%)	NS		9	(3%)
Renal malformations	166	(32%)	89	(45%)	85	(30%)	23	(20%)	92	(27%)	72	(31%)	36	(29%)	38	(34%)^e^	32	(13%)
Unilateral renal agenesis	NS		62	(31%)	53	(19%)	NS		44	(13%)	43	(17%)	19	(15%)	24	(22%)	17	7%
Pelvic/ectopic kidney	NS		10	(5%)	26	(9%)	NS		27	(8%)	13	(5%)	9	(7%)	7	(6%)	10	
Horseshoe kidney	NS		9	(5%)	3	(1%)	NS		1	(< 1%)	5	(2%)	1	(1%)	3	(3%)	0	
Duplex kidney	NS		8	(4%)	9	(3%)	NS		12	(3%)	NS		3	(2%)	5	(5%)	2	
Skeletal malformations	65	(12%)	18	(9%)	54	(19%)	6^a^	(5%)	71	(21%)	59	(32%)^c^	40	(32%)	21	(13%)	51	(41%)
Scoliosis	NS		11	(6%)	NS		NS		38	(11%)	NS		21	(17%)	NS		43	(34%)
Klippel-Feil anomaly	NS		3	(2%)	NS		NS		3	(1%)	9	(5%)	4	(3%)	NS		NS	
Hemivertebrae	NS		4	(2%)	NS		NS		NS		NS		NS		NS		NS	
Other	NS		NS		NS		NS		14	(4%)	NS		20	(16%)	NS		18	(14%)
Cardiac malformations	6	(1%)	NS		10	(4%)	6	(5%)	NS		NS		6	(5%)	6	(4%)	4	(1%)
Hearing impairment	NS		9	(5%)	NS		4	(3%)	14	(4%)	NS		4	(3%)	3	(2%)	1	(< 1%)
Other rare features	Neurological anomaly, *n* = 7; inguinal hernia, *n* = 27				Neurological anomaly, *n* = 14.				Inguinal hernia, *n* = 54; AA, *n* = 3; VACTERL association, *n* = 1.				Inguinal hernia, *n* = 8		AA, *n* = 3; VACTERL association, *n* = 2; hypothelia, *n* = 1		AA, *n* = 5	

*Abbreviations*: *AA* anal atresia, *NS* not stated, VACTERL,

^a^The authors excluded scoliosis as a skeletal malformation

^b^Classification was possible in 232 patients

^c^Skeletal imaging were performed in 184 patients

^d^Fifty-three patients were not classified

^e^Renal examinations were performed in 111 of 168 patients

### Differential diagnosis

Several other diagnoses show similarities with MRKH syndrome. Vaginal agenesis is occasionally misinterpreted as imperforate hymen or transverse vaginal septum in which US exam will reveal a proximal vaginal canal and possibly hematocolpos. Such misinterpretations may have unfortunate iatrogenic implications [109]. A rare form of Müllerian agenesis in females is associated with clinical virilization/hyperandrogenemia and is caused by mutations in *WNT4* (OMIM #158330) and generally considered a separate entity from MRKH syndrome [49]. Müllerian agenesis is sometimes falsely reported in 46,XX and 45,X females with ovarian insufficiency (gonadal dysgenesis) and estrogen deficiency. However, in these patients exogenous exposure to estrogen have been reported to induce uterus development suggesting absent pubertal uterine development and not agenesis [110]. In general, imaging of the prepubertal uterus should be cautiously interpreted due to the risk of false positive conclusions of agenesis/aplasia.

Several features are also shared with various 46,XY disorders of sexual development. Complete androgen insensitivity syndrome (CAIS, also referred to as Morris syndrome) which is an X-linked disorder affecting genetically males (46,XY) caused by hemizygous mutations in the androgen receptor gene, *AR* (OMIM #300068). These patients have normal female appearance, blind-ending vagina and absent uterus and have breast development but sparse pubic hair at puberty. Also, 17-hydroxylase/17,20-lyase deficiency in 46,XY females caused by biallelic *CYP17A1* mutations include normal (or sometimes ambiguous) external genitalia, absent uterus and a shortened vagina due to incomplete masculinization (OMIM #202110) [111]. In doubt of these differential diagnoses, chromosomal analysis is useful and should be considered in order to differentiate MRKH syndrome from 46,XY DSDs.

### Psychological and psychosexual issues in MRKH syndrome

The diagnosis of MRKH syndrome may have profound psychological and/or psychosexual impact and it is a hallmark in the management to counsel the patient and support mental health upon the diagnosis and onwards in life [82, 112, 113]. Receiving the diagnosis, many patients experience facing overwhelming issues regarding identity, sexuality and infertility, and the importance of good caring and counselling should not be underestimated. The diagnosis is often made during adolescence; a period of sensitive physical/emotional development and vulnerability, which further imposes the provider’s caring and awareness towards the patients’ emotions, reactions and coping strategies. Furthermore, it is important to be aware of potential cultural aspects and their influence on reactions to the diagnosis in patients and their families and peers.

Knowledge of the psychological aspects in MRKH syndrome remains limited but studies have agreed on presence of higher levels of psychological distress in patients compared with women without MRKH syndrome [114, 115]. Most studies have been focusing on sexual function and well-being using quantitative survey testing such as Female Sexual Functioning Index (FSFI) and Female Sexual Distress Scale-Revised (FSDS-R), which particularly have been used measuring functional outcome following neovaginal therapy [116–118]. Other studies have also focused on specific psychological/psychiatric symptoms such as self-esteem, depression and anxiety [114, 115, 119, 120]. Bargiel-Matusiewicz et al. reported increasingly higher neuroticism following diagnosis as well as low level of problem-focused coping style [121]. To our knowledge only two qualitative studies on psychological issues in MRKH syndrome have been published providing valuable insight to the lived experience of having MRKH syndrome [122, 123]. Ernst et al. reported on motivators and barriers to disclosure, emotions receiving the diagnosis and its impact onwards in life [122]. Patterson et al. identified four major themes having MRKH syndrome: hindered independence in relation to the mother’s involvement in the care, feelings of being different, difficulties managing intimacy, and the threat to female identity [123].

Sexuality and sexual activity are complex concepts which should be viewed in the broadest senses and not merely limited to the mechanistic ability to engage penetrative intercourse [124]. The World Health Organization (WHO) suggests a holistic approach describing sexuality in biopsychosocial terms. In this view, it is important to recognize the patient’s possibilities and abilities to reach good self-esteem and well-being, establish sexual relations, and empower the patient with coping capabilities to navigate through the psychosexual challenges which may follow having MRKH syndrome [115]. While recognizing the challenge for any health-care professional to provide sufficient counselling and support, referral to experts in genital malformations and/or disorders of sexual development, sexologists or psychologists is often relevant and should always be considered.

Many patients experience difficulties talking about feelings and emotions related to MRKH syndrome. Therefore, patients should be encouraged to gain trust with someone close (parents, partner, friend) with whom thoughts can be shared [122]. Sharing thoughts and emotions with other patients can be valuable and group programs for women with MRKH syndrome have been reported to decrease psychological distress [125, 126]. Patients should also be encouraged to connect to a peer support group allowing them to learn from other patients’ experiences. There are several support groups established worldwide (see list at ref. [127]).

### Management of vaginal agenesis

Historically, correction of vaginal agenesis in MRKH syndrome with creation of a functional neovagina has been a hallmark in the treatment. During the last hundred years, various different surgical and non-surgical methods have been suggested for vaginal construction. Surgical procedures include vaginoplasties using various autografts such as McIndoe vaginoplasty (split-skin graft covering a mold placed in the dissected pouch between the rectum and bladder), Baldwin vaginoplasty (bowel graft), Davydov vaginoplasty (peritoneal graft), and Williams vulvavaginoplasty (labia majora flaps) [128–131]. More recently, vaginoplasties using cultured autologous vulvar tissue [132, 133] and tissue-engineered biomaterial [134] have been suggested. An alternative surgical procedure is the laparoscopic Vecchietti vaginoplasty in which a surgical traction device is placed on the anterior abdominal wall with subperitoneal threads attached to a mold in the vagina [135, 136]. The most commonly used non-invasive method is self-dilation (also referred to as Frank’s method) [137] In this method, progressive dilators are manually placed on the vaginal apex for 10–30 min one to three times a day. Thorough counseling to ensure patient readiness and firm instructions in the technique should always precede start of dilation. Therapeutical education throughout the treatment is pivotal to achieve success and the patient should be followed closely in order to monitor progress and provide good support and guidance. An alternative non-invasive option is dilation by intercourse (d’Alberton’s method) [138] which has been reported with good anatomical and functional outcome compared with self-dilation and surgery [118, 139]. This method, however, requires regular coital activity with a partner and thus, this approach is not an option for all patients. Importantly, the three latter methods, which are based on dilation of the vaginal dimple, will provide the vagina with a normal mucosal lining. This may be advantageous in a uterus transplantation situation (see below) since this will provide the vagina with a normal vaginal microbiota, which may be of importance for success at embryo transfer as well as for correctly grade rejection by cervical biopsy.

A comprehensive literature review on the management of vaginal agenesis has been conducted by Callens et al. reviewing outcome, advantages and disadvantages of the different procedures [140]. In general, disadvantages of the surgical methods are the invasiveness and need of anesthesia, the risk of neovaginal strictures requiring onwards dilation following surgery as well as specific complications related to different graft tissues. Disadvantages in dilation therapy include the risk of low compliance (especially in younger patients), time consume needed for a satisfactory result, the discomfort that some patients experience, and a low risk of urethral dilation [139, 140]. Discussing different treatment options with the patient, it is important to emphasize that there is no quick solution to obtain a functional vagina and surgical options still require continued postoperative dilation, by regular intercourse or vaginal dilators, to ensure satisfactory long-term outcome.

Since 2002, The American College of Obstetricians and Gynecologists (ACOG) has recommended dilation therapy as first line treatment based on the high overall success rate (90–96%), being non-invasive with a low complication rate, and low costs [82, 141–143]. Due to the risk of low compliance using dilators, the treatment should be supervised and followed by a health professional experienced in this therapy. ACOG recommends that surgery should be reserved for patients experiencing failure with dilation therapy and emphasizes that surgery still requires post-surgical dilation to avoid strictures. Dilation therapy as first choice is also supported by Callens et al. [140], which further suggest laparoscopic Vecchietti vaginoplasty as preferred second-line therapy. Most importantly, thorough counseling regarding expected outcome and possible complications should always precede any attempt for vaginal construction, and it is fundamental to ensure the full maturity and motivation of the patient undergoing such treatment. Moreover, it is important to recognize the option of no treatment, which for some patients might be the right choice [144].

Despite of the accumulated literature and the ACOG recommendation, dilation as first-line therapy for vaginal agenesis is still not widely accepted by surgical experts in the field. The rarity of the syndrome implies that most treating centers only acquire expertise and thus preference for a single procedure. This may result in reporting and publication biases in the available literature concerning outcome and complications and it complicates the initiation of comparative studies including both surgical and non-surgical approaches. Furthermore, most studies differ in terms of follow-up and measuring outcome [140]. Only few comparative studies have been reported to date [104, 118, 139, 145–150] and those studies comparing dilation therapy with surgical procedures generally conclude dilation to be non-inferior to surgery [104, 118, 139, 145, 146, 148, 150]. Another common limitation in most previous studies is the focus only on anatomical outcome (neovaginal depth and width) instead of functional outcome and patient satisfaction [151]. Fortunately, this seems to have gained focus in the contemporary literature. In conclusion, prospective studies based on therapeutical education within multidisciplinary expert teams using standardized outcome measurements of both anatomical and functional results and complications with long-term follow-up are highly requested in order to ascertain these preliminary reports for future clinical recommendations.

### Infertility and uterus transplantation (UTx)

Women with MRKH syndrome belong to the group of females with absolute uterine factor infertility (AUFI), which comprise those with anatomical absence of a uterus or presence of a non-functional uterus, in terms of implanting an embryo and carrying a pregnancy. Other groups of AUFI that lack a uterus, apart from MRKH syndrome, are women hysterectomized during fertile life because of malignancy (mainly cervical cancer), benign disease (leiomyoma) or as a life-saving procedure because of massive obstetrical bleeding. Women with intrauterine adhesions, large inoperable leiomyoma, or some specific uterine malformations also belong to the group of women with AUFI.

The motherhood options for women with MRKH syndrome, and other causes of AUFI, have traditionally been legal adoption, as international or national adoption. Since the mid-1990s gestational surrogacy (GS), has been an alternative to gain genetic motherhood, and after adoption from the birth-giving mother, also legal motherhood. In this procedure, in vitro fertilization is first performed with oocytes from the MRKH woman and her partner’s sperm. An embryo is then placed inside the womb of another women, the gestational surrogate carrier. The GS arrangement may be commercial or altruistic (typically with a close relative (mother, sister) as the carrier) depending on jurisdiction in the specific nation/state, as exemplified by the difference in arrangements in California, with mainly commercial GS, and Canada with exclusively altruistic GS [152]. Gestational surrogacy is not allowed in the Nordic countries and in most other parts of the world, the reasons being ethical, religious or legal or a combination of these [153]. However, it is well-known that GS is used by many women with MRKH syndrome, residing in countries with non-approval of GS, and concerning Northern Europe the main countries for reproductive tourism concerning GS seem to be USA, Ukraine and Georgia. Until recently India was the main GS country for Nordic couples with female AUFI, but India has now banned commercial GS [154].

Uterus transplantation (UTx) has now emerged as the first true infertility treatment for women with MRKH syndrome and giving them full (gestational, genetic, legal) motherhood from start. The group in Gothenburg, Sweden, initiated basic UTx research in animal models already in 1999. The idea to this concept, was given by a young woman, with cervical cancer, who would undergo a radical hysterectomy [155]. Through structured animal-based research [156] and continuous ethics discussions [157] within the research group in Sweden the team optimized the surgical technique, immunosuppression for a uterine graft, rejection diagnosis as well as assuring normal development of the offspring from allogeneic UTx [158]. In 2012, the team received permission from the ethics board as well as permission from the hospital board to perform a prospective observational study of live donor UTx, including up to ten procedures. The surgeries, performed in 2013, included eight MRKH syndrome patients and one cervical cancer patient as recipients. The donors were five mothers, one sister, one maternal aunt, one mother-in-law and one family friend [159]. The surgical outcome of the nine UTx procedures was that donor surgery was extremely difficult, with surgical durations of 10–13 h, but that the transplantation procedures in the recipients were simpler, with surgical durations of 4–5 h. Seven out of nine procedures were surgically successful, with viable grafts showing regular menstruations during the first post-transplantation year. Two out of the seven grafts had to be surgically removed during the initial months, because of vascular thrombosis in one case and intrauterine infection in the other case [159].

The proof-of-concept of UTx as an infertility treatment to women with MRKH came with the world’s first livebirth after UTx which took place in September, 2014 in Gothenburg, Sweden [160]. Patient number 5, in the cohort of nine women in the original Swedish UTx study [159], had MRKH syndrome with also unilateral kidney agenesis. She had acquired a neovagina through self-dilation and underwent UTx at age 35 years, receiving a uterine graft from a 61 year-old, 2-parous, family friend that had been postmenopausal for 7 years before uterus donation. One year after UTx she underwent transfer with a single embryo and achieved pregnancy, which continued essentially uneventful until the recipient acquired preeclampsia in gestational week 31 + 5, with cesarean section performed the following day. A healthy boy was delivered and his development since then has been normal.

This birth was followed by seven more live births in Sweden [161, 162], until the ninth UTx baby in the world was born in USA in December 2017, after a live donor UTx procedure [163]. There also exist several births after deceased donor UTx [164]. Today, approximately 75 UTx procedures have been performed and all but two of these have been performed in MRKH patients (Brännström, personal communication). Around 25 babies have been born worldwide, with some of the MRKH patients having delivered healthy babies twice.

**Fig. 2:**
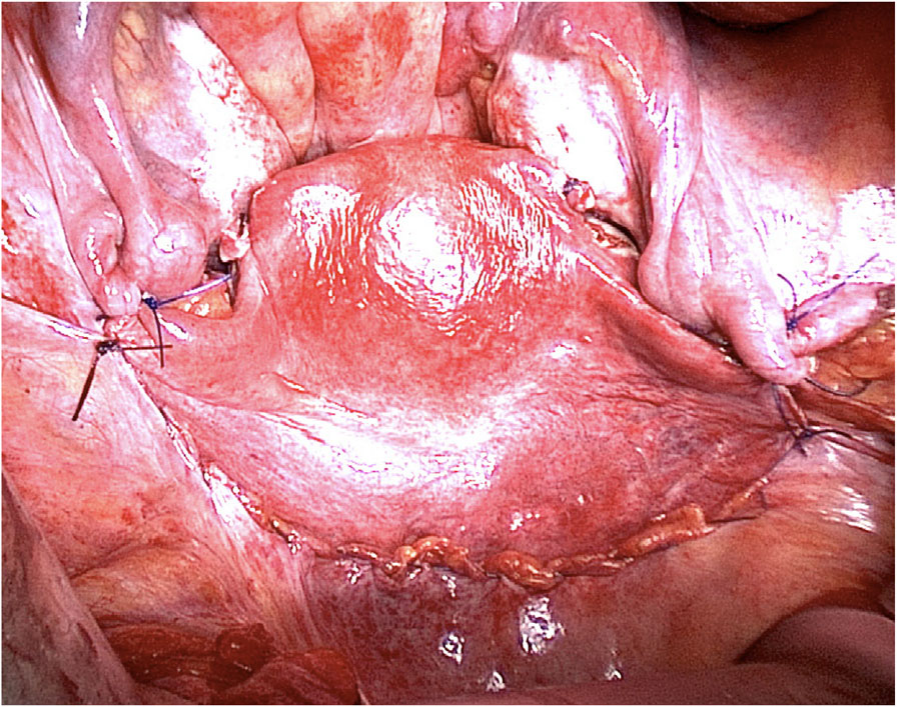
A transplanted uterus in the pelvis of a woman with MRKH syndrome. The uterus is seen in the middle. The bladder is seen below the uterus and the rectum is above

The UTx surgery of the MRKH women (Fig. 2) is generally preformed through a sub-umbilical midline incision. The vaginal vault is dissected free from the bladder and the rectum and this is possible through cleavage of the uterine remnant in the midline. The external iliac arteries and veins are then dissected free and cleaned from surrounding tissue, to allow for anastomosis. Brännström and his team usually also attach fixation sutures to the sacrouterine ligaments, round ligaments and to the cardinal ligaments. The chilled and flushed uterus is then coming from the back-table into the pelvis. End-to-side anastomoses are then performed with the internal iliac segments on the uterine vessels of the graft to the external iliac vessels of the recipient. After reperfusion, the vagina is opened to perform end-to-end vaginal anastomosis, which then is followed by fixation of the uterus to the ligaments.

The immunosuppression used for the transplanted patients is standard induction therapy, similar to what is used for kidney transplantation. The mainstay of maintenance immunosuppression has been the calcineurin inhibitor, tacrolimus [162].

In the future it is likely that minimal invasive surgery, by robotic-assisted laparoscopy, will be the main surgical approach for live donor surgery and some years later also for recipient surgery in UTx [165]. Advantages for the patients will be less tissue trauma, with possibility to decrease the length of hospital stay to 1–2 days and sick-leave to 1–2 weeks.

It is important to point out that already at this early stage of human UTx, which is still at an experimental phase, the procedure has proved to be an effective fertility treatment with a take-home-baby rate above 80%, in patients with graft survival of more than six months after UTx (our unpublished observations). It is likely that the efficiency of UTx will become even greater in the future, in line with the advancement of other medical procedures. Another essential aspect of UTx, in comparison to other types of solid organ transplants, is that UTx is the first ephemeral transplantation, where immunosuppression is only for a restricted time, and the medication can be withdrawn when the graft is removed. This is typically accomplished within 5 years, when the transplanted woman has given birth to the desired number of children. Thus, the negative, long-term side effects of calcineurin inhibitors, such as nephrotoxicity, can be avoided. This is important in the context of MRKH syndrome, where several patients have single kidneys.

## Conclusions

The caring of patients with MRKH syndrome is complex and requires a patient-centered multidisciplinary approach in careful dialogue with the patient addressing all-together gynecological, sexual, psychological and infertility issues. Continuous research efforts and collaborations are pivotal in order to expand the current knowledge and improve future care. Fortunately, several advances are made these years across disciplines including the genetics of MRKH syndrome providing a better understanding of the etiology, improved diagnostics as well as optimal care and counselling. Ultimately, the advent of UTx as the first available fertility treatment for MRKH syndrome has provided new hope for these patients to become pregnant and achieve biological motherhood.

## Data Availability

Not applicable.

## Abbreviations

AA: Anal atresia
ACOG: American College of Obstetricians and Gynecologists
AMH: Anti-müllerian hormone
AUFI: Absolute uterine factor infertility
BRA: Bilateral renal agenesis
CAIS: Complete androgen insensitivity syndrome
CNV: Copy number variation
CT: Computed tomography
DSD: Disorder(s) of sexual development
FSDS-R: Female Sexual Distress Scale-Revised
FSFI: Female Sexual Functioning Index
FSH: Follicle stimulating hormone
GS: Gestational surrogacy
IVF: In vitro fertilisation
LH: Luteinizing hormone
MRI: Magnetic resonance imaging
MRKH: Mayer-Rokitansky-Küster-Hauser
MURCS: Müllerian duct aplasia-renal agenesis-cervicothoracic somite dysplasia
NGS: Next-generation sequencing
OMIM: Online Mendelian Inheritance in Man
PMD: Paramesonephric (Müllerian) duct
URA: Unilateral renal agenesis
US: Ultrasonography
UTx: Uterus transplantation
VACTERL: Vertebral defect, anal atresia, cardiac defect, tracheoesophageal fistula/esophageal atresia, renal defect, and limb defect
WES: Whole-exome sequencing
WGS: Whole-genome sequencing
WHO: World Health Organization
